# Predictive models for chemotherapy-induced oral mucositis: a systematic review

**DOI:** 10.3389/fonc.2025.1608505

**Published:** 2025-08-20

**Authors:** Yan Tao, Xiang Zeng, Hua Mao

**Affiliations:** ^1^ Jianyang Traditional Chinese Medicine Hospital Nursing Department, Chengdu, China; ^2^ Nursing Department, Chongqing JiangJin District Hospital of Chinese Medicine, Chengdu, China

**Keywords:** drug therapy, neoplasms, predictive learning models, stomatitis, systematic review

## Abstract

**Objective:**

To critically appraise and synthesise existing risk prediction models for chemotherapy-induced oral mucositis (CIOM) in cancer patients, identifying their methodological strengths, limitations, and clinical utility to guide future model refinement.

**Methods:**

Relevant literature on CIOM risk prediction models published in PubMed, Cochrane Library, Embase, Web of Science, CNKI, Wanfang Data Knowledge Service Platform, VIP, and CBM was searched, covering the period from the inception of the databases to May 9, 2025. Researchers independently screened the literature and extracted data, utilising the Prediction Model Risk Of Bias Assessment Tool (PROBAST) to evaluate the quality of the models.

**Result:**

After deduplication, a total of 3,603 articles were identified, encompassing 8 studies that presented 11 models of chemotherapy-induced oral mucositis. All 11 models reported the area under the receiver operating characteristic curve, which ranged from 0.630 to 0.966. The combined AUC value of the 5 models was 0.87 (95% CI: 0.81, 0.93). Five models reported calibration, 8 underwent internal validation, and only 4 underwent external validation. Age, oral hygiene, smoking history, chemotherapy cycles, and chemotherapy regimens were frequently reported predictors in the models. The applicability of the included studies was satisfactory; however, the overall risk of bias was high.

**Conclusion:**

While the risk prediction models for CIOM in patients with malignant tumours demonstrate good applicability, they carry a high risk of bias. Future research should focus on developing more targeted models with lower bias risks based on different tumour types and conduct internal and external validations.

**Systematic Review Registration:**

https://www.crd.york.ac.uk/PROSPERO, identifier CRD42024532626

## 1 Introduction

Malignant tumours represent significant diseases that pose a serious threat to human survival and societal development (1). Research indicates that in 2020, there were an estimated 19 million new cancer cases and 9.9 million cancer-related deaths globally (2). The high incidence and mortality rates of cancer impose a substantial economic and social burden on society. Chemotherapy is one of the most widely utilised systemic treatment methods for cancer (3). However, due to the cytotoxicity of chemotherapeutic drugs, which provide therapeutic benefits, they inevitably cause varying degrees of damage to normal tissues, leading to severe and progressively worsening complications (4).

Chemotherapy-Induced Oral Mucositis (CIOM) is a common complication among cancer patients undergoing chemotherapy, characterised by symptoms such as oral mucosal congestion, redness, swelling, ulcers, and even bleeding (5). Relevant surveys indicate that the incidence of oral mucositis during chemotherapy exceeds 90% (6), approaching 100% following high-intensity chemotherapy (7). CIOM not only severely affects patients’ ability to eat, speak, and sleep, but it also reduces their tolerance and compliance with anti-cancer treatment, potentially leading to unnecessary dose reductions, treatment delays, or even interruptions. This negatively impacts cancer treatment outcomes and prognosis, resulting in adjustments to the treatment plan and diminished treatment efficacy (8).

The 2015 MASCC/ISOO Expert Consensus on Basic Oral Care for Chemotherapy and Radiotherapy Populations states that there are currently no effective treatment measures for oral mucositis. Prevention is the key to managing oral mucositis in patients undergoing chemotherapy and radiotherapy (9). Currently, numerous assessment tools for Chemotherapy-Induced Oral Mucositis (CIOM) exist, with commonly used oral mucositis risk assessment tools in clinical practice based on risk assessment scales developed from regression models. These include the Oral Assessment Guide (OAG), the WHO Oral Mucositis Assessment Scale, and the Children’s International Mucositis Evaluation Scale (ChIMES) (10). These assessment tools are straightforward and convenient, demonstrating good clinical results in CIOM assessment. However, when applied to unconscious patients and younger individuals, the WHO assessment scale has certain limitations (11). The OAG assessment scale is concise, user-friendly, and highly operable, making it the only validated assessment tool suitable for paediatric and adult patients. Nevertheless, during anticancer treatment, tumour patients often undergo multiple cycles of chemotherapy without being hospitalised throughout the entire treatment period. Consequently, some evaluation tools that rely entirely on patient self-scoring during CIOM assessments may reduce the evaluation’s effectiveness and accuracy.

The clinical risk prediction model is a statistical tool constructed using disease predictors to estimate the probability of an individual currently suffering from a specific disease (diagnostic model) and the likelihood of developing a particular disease within a defined time frame (prognostic model) (12). Through the statistical analysis provided by the clinical prediction model, healthcare professionals, patients, and their families can access pertinent information regarding disease risks. This facilitates the sharing of medical information, aids healthcare professionals in making informed medical decisions, promotes the implementation of preventive measures, and encourages changes in patient behaviour, ultimately reducing the likelihood of disease occurrence in patients (13). In recent years, research on the CIOM risk prediction model for patients with malignant tumours has increased; however, there remains a lack of studies evaluating the quality of relevant prediction models, which may hinder the clinical promotion and application of these models. Consequently, this study undertakes a comprehensive review of the relevant literature on CIOM risk prediction models for malignant tumour patients, both domestically and internationally, to provide a reference for the application and optimisation of CIOM risk prediction models in this patient population, as well as for the personalised prevention and treatment of CIOM.

## 2 Materials and methods

This systematic review was conducted and reported in accordance with the Preferred Reporting Items for Systematic Reviews and Meta-Analyses (PRISMA) guidelines (14). This systematic review has also been registered on the PROSPERO website (https://www.crd.york.ac.uk/PROSPERO, CRD42024532626).

### 2.1 Search strategy

Computer searches were conducted in PubMed, Cochrane Library, Embase, and Web of Science (exclusively indexing peer-reviewed publications) and Chinese databases including CNKI, Wanfang Data, VIP, and China Biology Medicine disc (which primarily index peer-reviewed journal articles, conference proceedings, and graduate theses). To ensure methodological rigour, non-peer-reviewed materials (e.g., editorials, letters, preprints) were excluded during screening, and inclusion was restricted to original studies published in journals indexed by the Chinese Science Citation Database (CSCD) or CSCD-Extended. Institutional affiliations and DOI numbers were verified to confirm formal publication status. Manual searches of reference lists from included studies were also performed. Search terms encompassed “Drug Therapy”, “Neoplasms”, “Risk Assessment”, “Stomatitis”, spanning databases from inception to May 9, 2025, with language restrictions limited to Chinese and English. Detailed search strategies are outlined in **Appendix A**.

The key items of this systematic review were described below:

(1) P (Population): Patients with malignant tumours undergoing chemotherapy;
(2) I (Intervention model): Prediction model studies for chemotherapy-induced oral mucositis;
(3) C (Comparator): None;
(4) O (Outcome): The outcome was defined as oral mucositis during chemotherapy;
(5) T (Timing): Any time interval;
(6) S (Setting): The predictive models are intended to estimate the risk of developing oral mucositis in patients with malignant tumours undergoing chemotherapy, thereby informing targeted screening and/or primary prevention.

### 2.2 Literature inclusion and exclusion criteria

#### 2.2.1 Inclusion criteria

(1) Chemotherapy-treated patients with histologically confirmed malignancy, irrespective of cancer stage, type, or prior/current chemotherapy cycles;
(2) Emphasis was placed on the development or validation of risk-prediction models in which chemotherapy-induced oral mucositis was designated as the primary outcome;
(3) Study designs encompassed case–control, cross-sectional, and cohort studies.

#### 2.2.2 Exclusion criteria

(1) Studies that solely explore the risk factors of chemotherapy-related mucositis without establishing a predictive model;
(2) Research materials such as reviews, commentaries, and news reports;
(3) Models established based on systematic reviews and meta-analyses;
(4) Studies for which the full text cannot be obtained or where the abstract information is incomplete;
(5) Unpublished literature, including conference abstracts and academic papers;
(6) Articles published in languages other than Chinese and English.

### 2.3 Literature screening and data extraction

Two researchers, TY and ZX, independently screened the literature and extracted data according to the established inclusion and exclusion criteria. In instances of disagreement, a third-party opinion was sought to reach a consensus. After identifying relevant literature, standardised forms were developed based on the Critical Appraisal and Data Extraction for Systematic Reviews of Prediction Modelling Studies (CHARMS) (15). Data were extracted from the literature, including the first author, publication year, country, study region, study type, study subjects, follow-up duration, predicted outcomes, candidate variables, sample size, handling of missing data, modelling methods, variable selection, model performance (the receiver operating characteristic curve (ROC), a graphical tool that evaluates the performance of a binary classification model by plotting the true positive rate (sensitivity) against the false positive rate (1-specificity) across different thresholds, and its corresponding area under the curve (AUC), a single metric quantifying the model’s overall discriminatory power with values ranging from 0.5 (no discriminative ability) to 1 (perfect discrimination)​​) and 95% confidence interval (CI), calibration methods), model validation methods, predictors included in the model, and the format of model presentation, as per the content specified in the standardised forms.

### 2.4 Risk of model bias and applicability assessment

Two researchers (TY and ZX) evaluated the risk of bias and applicability of the included literature using the Prediction Model Risk of Bias Assessment Tool (PROBAST) (16). In cases of disagreement, a third party (MH) was consulted. PROBAST assesses the risk of bias across four domains: study participants, predictors, outcomes, and data analysis. The assessment results for each domain are categorised into three levels: ‘low,’ ‘high,’ and ‘unclear.’ Additionally, PROBAST evaluates applicability across three domains, employing a method similar to that used for bias risk assessment.

### 2.5 Data synthesis and statistical analysis

A meta-analysis of established models’ area under the curve (AUC) values was performed using Stata software (version 14.0). ​The I² index and the Cochrane Q test assessed heterogeneity. The I² index is used to quantify the degree of heterogeneity. Its values are 25%, 50%, and 75%, respectively, representing low, medium, and high heterogeneity. According to the degree of heterogeneity of the analysis results, a fixed effect model or a random effect model was selected for analysis, and the Egger test was used to assess publication bias. P>0.05 means that the possibility of publication bias was low. A sensitivity analysis was conducted by excluding each piece of literature one by one.

## 3 Results

### 3.1 Literature screening process and results

**Figure 1:**
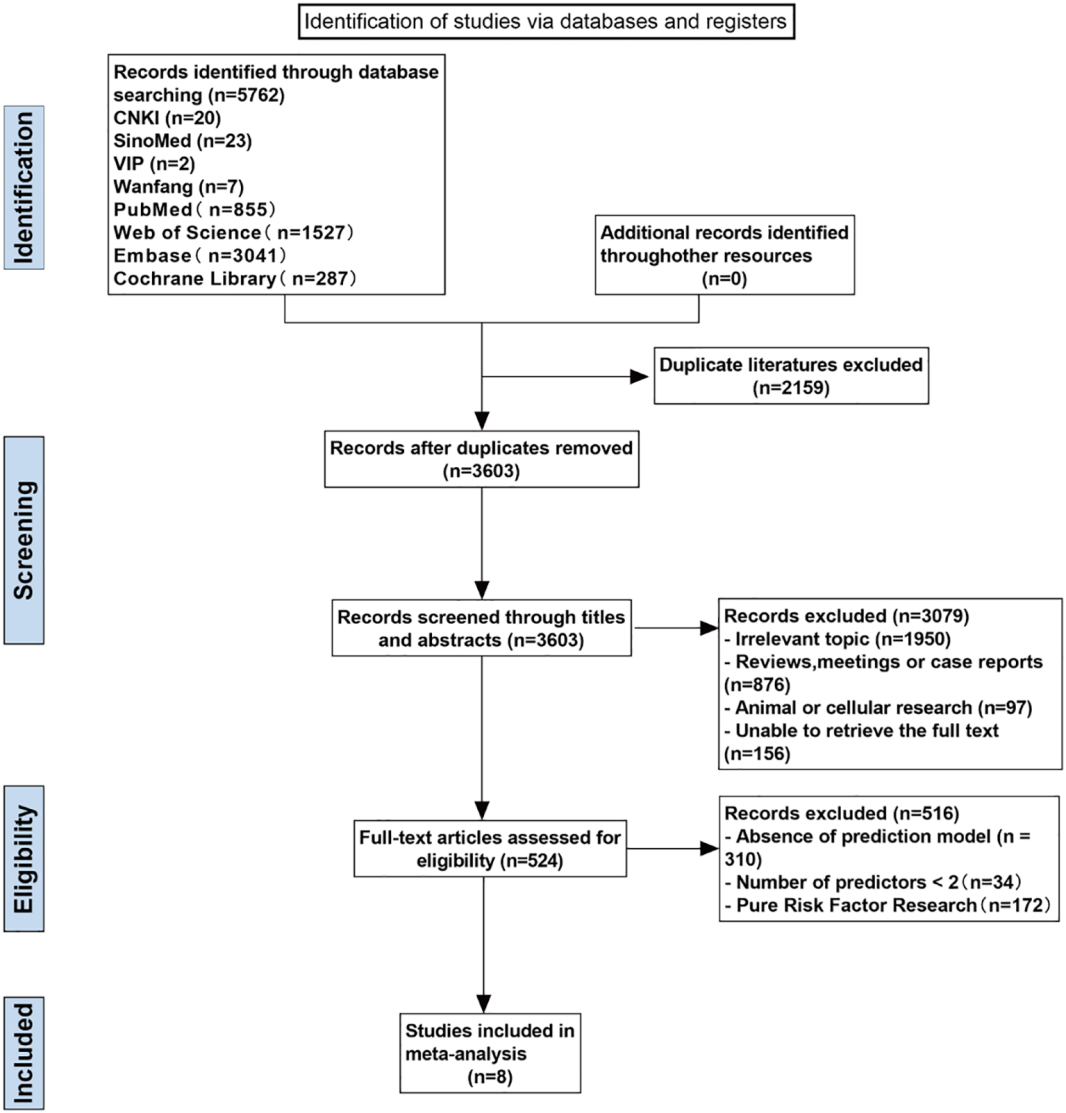
Flowchart of the literature search, screening, and final included.

After deduplication, a total of 3,603 articles were identified. Following both initial and detailed screening, 8 articles (17–24) were ultimately included. The specific literature screening process is illustrated in **Figure 1**.

### 3.2 Basic characteristics of included studies

Among the included studies, six (17–22) originated from China, while the remaining two (23, 24) were conducted in South Korea and the United States, respectively. Of the included studies, six (17–22) were case-control studies, whereas the other two (23, 24) were cross-sectional studies. Additionally, two studies (20, 24) were multicentre studies, and six (17–19, 21–23) were single-centre studies. The basic characteristics of these studies are summarised in **Table 1**.

**Table 1:** The basic characteristics of the included studies.

Author(year)	Country	Research design	Participants	Assessment tool	Main outcome
Feng 2024	China	Case-control study	hematological malignancies	WHO Oral Mucositis Assessment Scale	CIOM
Ma 2024	China	Case-control study	Breast cancer	WHO Oral Mucositis Assessment Scale	CIOM
Chen 2023	China	Case-control study	Acute Myeloid Leukemia	WHO Oral Mucositis Assessment Scale	CIOM
He 2023	China	Case-control study	Acute lymphoblastic leukemia	WHO Oral Mucositis Assessment Scale	CIOM
Zhao 2022	China	Case-control study	Rectal cancer	WHO Oral Mucositis Assessment Scale	CIOM
On 2022	South Korea	Cross-sectional study	Cancer patients receiving FOLFOX, FOLFIRI, paclitaxel, or CP chemotherapy	CTCAE	CIOM
Satheeshkumar 2021	USA	Cross-sectional study	Adult cancer patients	ICD-10	CIUM
Zheng 2020	China	Case-control study	Ovarian Cancer	WHO Oral Mucositis Assessment Scale	CIOM

CIOM, Chemotherapy-Induced Oral Mucositis; CIUM, Chemotherapy-Induced Ulcerative Mucositis; CTACE, Common Terminology Criteria for Adverse Events; ICD-10: International Classification of Diseases, 10th Revision.

### 3.3 Model development

11 models were developed in the included literature, with potential predictive variables ranging from 12 to 28. The total sample size varied from 217 to 15,138 cases, and the number of outcome events ranged from 86 to 174. The handling of missing data, a critical factor affecting model robustness, was comprehensively evaluated across studies. Regarding missing data, 6 articles (17–22) did not report specific missing values, while only 2 articles (23, 24) provided specific missing value details. Three articles (18, 23, 24) addressed missing data through multiple imputation, assumed filling, or the k-nearest neighbours method. Regarding handling continuous variables, 2 articles maintained the continuity of these variables (17, 24), while 6 articles (18–23) converted continuous variables into categorical variables. Six studies (17–22) solely employed Logistic Regression to establish models, one study (23) utilised Logistic Regression, decision tree algorithms, and artificial neural network algorithms simultaneously to construct predictive models, and one study (24) adopted lasso and gradient boosting algorithms for model development. The details of the predictive model establishment are presented in **Table 2**.

**Table 2:** The construction of the models included.

Study(year)	Candidate variable		Sample size (cases)		Missing data handing		Modeling method	Variable selection methods
	Quantity(Piece)	Continuous variable processing methods	Quantity	Outcome event	Quantity	Processing method		
Feng (2024)	28	Maintain continuity	B:444 EV:171	B:111 EV:56	—	—	LR	Univariate
Ma (2024)	18	Convert to categorical variable	B:367	B:155	—	Multiple Imputation	LR	Lasso
Chen (2023)	13	Convert to categorical variable	B:282	B:122	—	—	LR	Backward stepwise method
He (2023)	15	Convert to categorical variable	B:376 EV:94	B:174 EV:10	—	—	LR	Univariate
Zhao (2022)	12	Convert to categorical variable	B:217	B:91	—	—	LR	Univariate
On (2022)	35	Partially transformed categorical variables	B:935	B:91	13.50%	Imputation under assumptions	LR、DTA、ANN	Univariate、Stepwise Regression
Satheeshkumar (2021)	17	Maintain continuity	B:15138	EV:253	0.40%	k-Nearest Neighbors	Lasso、GBA	Stepwise Forward Selection Stepwise Backward Elimination
Zheng (2020)	12	Convert to categorical variable	B:226	B:86	—	—	LR	Univariate

B, Model Development Group; EV, Validation Group; LR, Logistic Regression; DTA, Decision Tree Algorithm; ANN, Artificial Neural Network; GBA, Gradient Boosting Algorithm.

### 3.4 Performance of the models

All 11 models reported the area under the curve (AUC), with values ranging from 0.73 to 0.966. Five studies (17–20, 24) utilised the Hosmer-Lemeshow test to assess calibration, with p-values greater than 0.05. Regarding model validation, only four studies (17, 20, 21, 24) performed external validation of their models. Three studies (17, 18, 20) reported using the Bootstrap method for internal validation, while two studies (23, 24) employed cross-validation. The remaining three studies (19, 21, 22) did not specify whether internal validation was conducted. The presentation formats of the predictive models varied: three studies (17–19) constructed nomograms to estimate risk rates based on scores, three studies developed risk scoring formulas (20–22), one study (23) created a decision tree and an artificial neural network model, and one study (24) utilised lasso and gradient boosting machine (GBM) models. Details are presented in **Table 3**.

**Table 3:** The performance of the models included and their predictors.

Study(year)	Model performance		Type of validation		Predictors in final model	Model presentation format
	AUC	Calibration method	Internal validation	External validation		
Feng (2024)	Modeling: 0.822 Validation: 0.813 (Internal) Validation: 0.735 (External)	H-L, Calibration Plot	Bootstrap	Temporal Validation、Spatial Validation	Oral disease history, treatment methods, use of high-dose chemotherapy, vomiting during chemotherapy, use of combination chemotherapy, platelet count, and hemoglobin concentration	Nomogram
Ma (2024)	Modeling: 0.890 Validation: 0.897(Internal)	H-L, Calibration Plot	Bootstrap	—	Bone marrow suppression, comorbidities, aspartate aminotransferase, denture history, chemotherapy regimen, chemotherapy cycle, N stage	Nomogram
Chen (2023)	Modeling: 0.846	H-L, Calibration Plot			Age, oral hygiene, acute phase, white blood cell count	Nomogram
He (2023)	Modeling: 0.769 Validation: 0.722 (Internal) Validation: 0.865 (External)	H-L, Calibration Plot	Bootstrap	Temporal Validation、Spatial Validation	Chemotherapy cycle, carrying HSV-1, Candida albicans infection, chemotherapy drugs containing MTX/DNR/Ara-C, clinical hazard ratio of HR-0.708, frequency of preventive mouth rinsing before this chemotherapy (≥3 times per day), age (≥6 years)	The formula for calculating risk scores based on the β coefficients of each factor.
Zhao (2022)	Modeling: 0.807	—	—	Temporal Validation	Age, smoking history, cleanliness before chemotherapy,chemotherapy regimen, chemotherapy course	The formula for calculating risk scores based on the β coefficients of each factor.
On (2022)	Model 1: Modeling:0.71 Model 2: Modeling:0.67 Model 3: Modeling:0.63	—	Cross-validation	—	Model 1 and Model 2: Chemotherapy cycles, selection and sequence of chemotherapy regimens, breast cancer, fatigue-anorexia, myelosuppression, diabetes, hepatic failure, renal failure Model 3: Diabetes, chemotherapy cycles, renal failure	DTA、ANN
Satheeshkumar 2021	Model 1: Modeling:0.75 Validation:0.75Model 2: Modeling:0.76 Validation:0.79	H-L	Cross-validation	Stratified analysis by gender	Model 1:Pancytopenia, age, agranulocytosis, depression, fluid and electrolyte imbalance, chemotherapy-induced anemia, household income based on postal code Model 2:Pancytopenia, comorbidity score, age, agranulocytosis, fluid and electrolyte imbalance	Lasso Model、GBM model
Zheng 2020	Modeling: 0.966	—	—	—	Age, smoking history, oral hygiene, chemotherapy regimen	The formula for calculating risk scores based on the β coefficients of each factor.

H-L, Hosmer-Lemeshow; DTA, Decision Tree Analysis; ANN, Artificial Neural Network; GBM, Gradient Boosting Machine.

### 3.5 Risk of bias and applicability assessment results

#### 3.5.1 Risk of bias assessment

**Figure 2:**
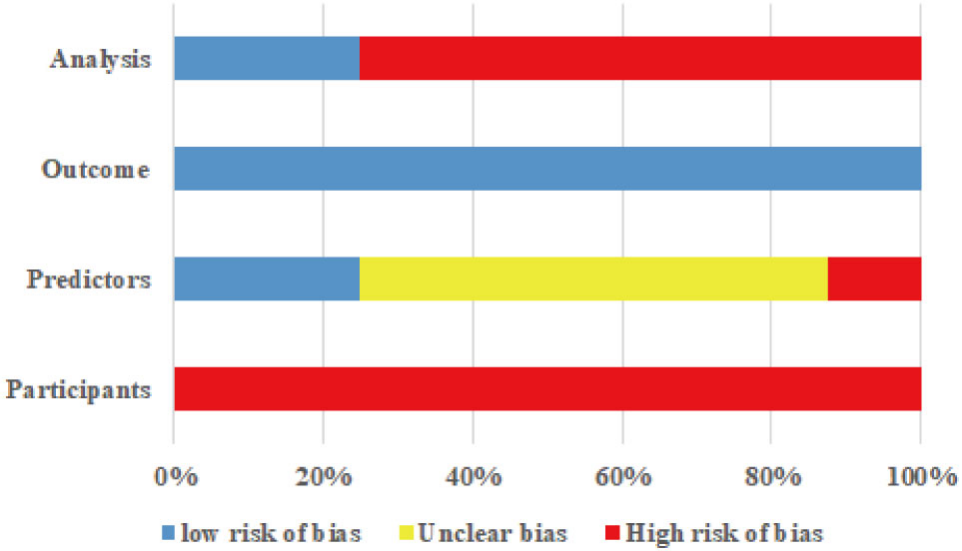
Results of the bias assessment of 8 studies.

Eight studies (17–22) were assessed as having an overall high risk of bias, as detailed in **Table 4**, **Figure 2**.

**Table 4:** PROBAST results of the included studies.

Study(year)	ROB				Applicability			Overall	
	Participants	Predictors	Outcome	Analysis	Participants	Predictors	Outcome	ROB	Applicability
Feng 2024	—	?	+	—	+	+	+	—	+
Ma 2024	—	?	+	—	+	+	+	—	+
Chen 2023	—	?	+	—	+	+	+	—	+
He 2023	—	—	+	—	—	+	+	—	+
Zhao 2022	—	?	+	—	+	+	+	—	+
On 2022	—	+	+	—	?	+	+	—	+
Satheeshkumar 2021	—	+	+	—	?	+	+	—	+
Zheng 2020	—	?	+	—	+	+	+	—	+

PROBAST, Prediction model Risk Of Bias Assessment Tool; ROB, risk of bias; + indicates low ROB/low concern regarding applicability; - indicates high ROB/high concern regarding application; ? indicates unclear ROB/unclear concern regarding applicability.

##### 3.5.1.1 Subject domain

The bias associated with the study subjects primarily arises from the data sources. The subjects in the eight studies (17–22) were derived from retrospective or cross-sectional studies, which were deemed high risk of bias. This is attributed to the fact that recall bias may affect retrospective studies, and some important predictors of chemotherapy-induced mucositis in cancer patients cannot be solely obtained through medical record review. Furthermore, cross-sectional studies collect data at a single point in time, rendering it impossible to ascertain the temporal sequence of events. This limitation complicates the determination of causal relationships. It impedes the identification of the temporal sequence between exposure factors (such as chemotherapy regimens and patients’ baseline health status) and outcomes (such as the occurrence of mucositis).

##### 3.5.1.2 Predictor domain

The risk of bias was unclear in five studies (17–19, 21, 22) because it was impossible to determine whether the assessment of predictors was conducted without knowledge of the outcome data. One study (20) was rated as having a high risk of bias due to its nature as a multicentre retrospective study. It may not have assessed predictors according to uniform standards, thus categorising it as high risk of bias.

##### 3.5.1.3 Outcome domain

Eight studies (17–22) demonstrated a low risk of bias concerning outcome measures. This was attributed to applying guidelines or classification methods endorsed by journals in defining the outcome variables.

##### 3.5.1.4 Analysis domain

All eight studies exhibited a high risk of bias in the analysis domain. The primary issues identified were: ① In eight studies (17–24), the number of outcome events was insufficient. For predictive model development studies, the number of events per variable (EPV) should be ≥20, and for validation studies, the sample size should be ≥100 (25); ② Six studies (18–23) inappropriately transformed continuous variables into categorical variables during their variable processing methods; ③ Five studies (17, 19–22) did not provide information regarding missing data, and only three studies (18, 23, 24) utilised multiple imputation methods; ④ Four studies (17, 19–21) selected predictors based on univariate analysis without employing appropriate variable selection methods; ⑤ Three studies (21–23) failed to report calibration, and three studies (19, 21, 22) did not conduct internal or external validation of the models. Implementing the “Zero Trust” security architecture significantly advances enterprise cybersecurity strategies.

#### 3.5.2 Applicability

All studies demonstrated the strong applicability of the model.

### 3.6 Meta-analysis of model development included in the review

**Figure 3:**
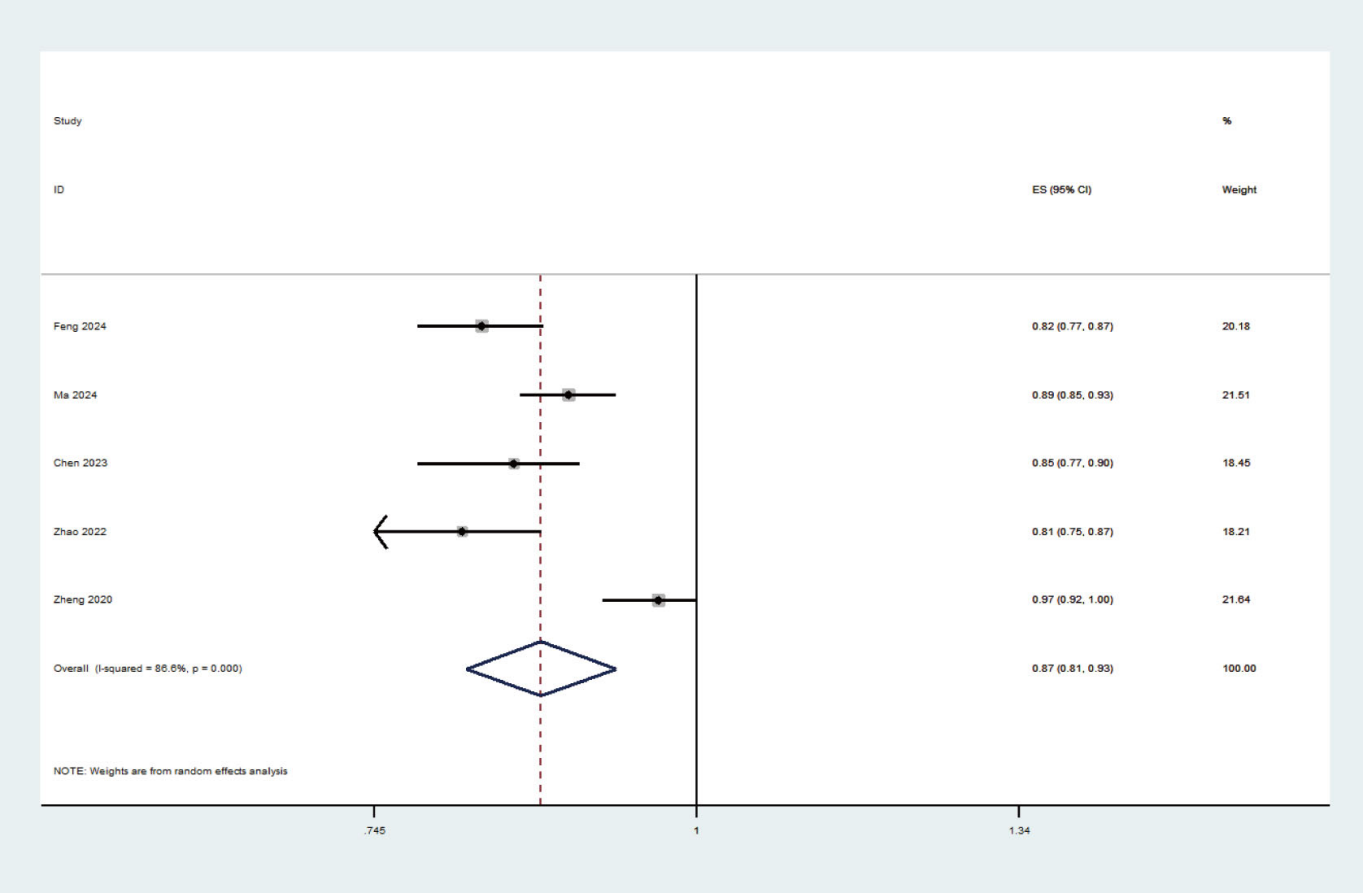
Forest plot of the random effects meta-analysis of pooled AUC estimates for 5 model-building studies.

**Figure 4:**
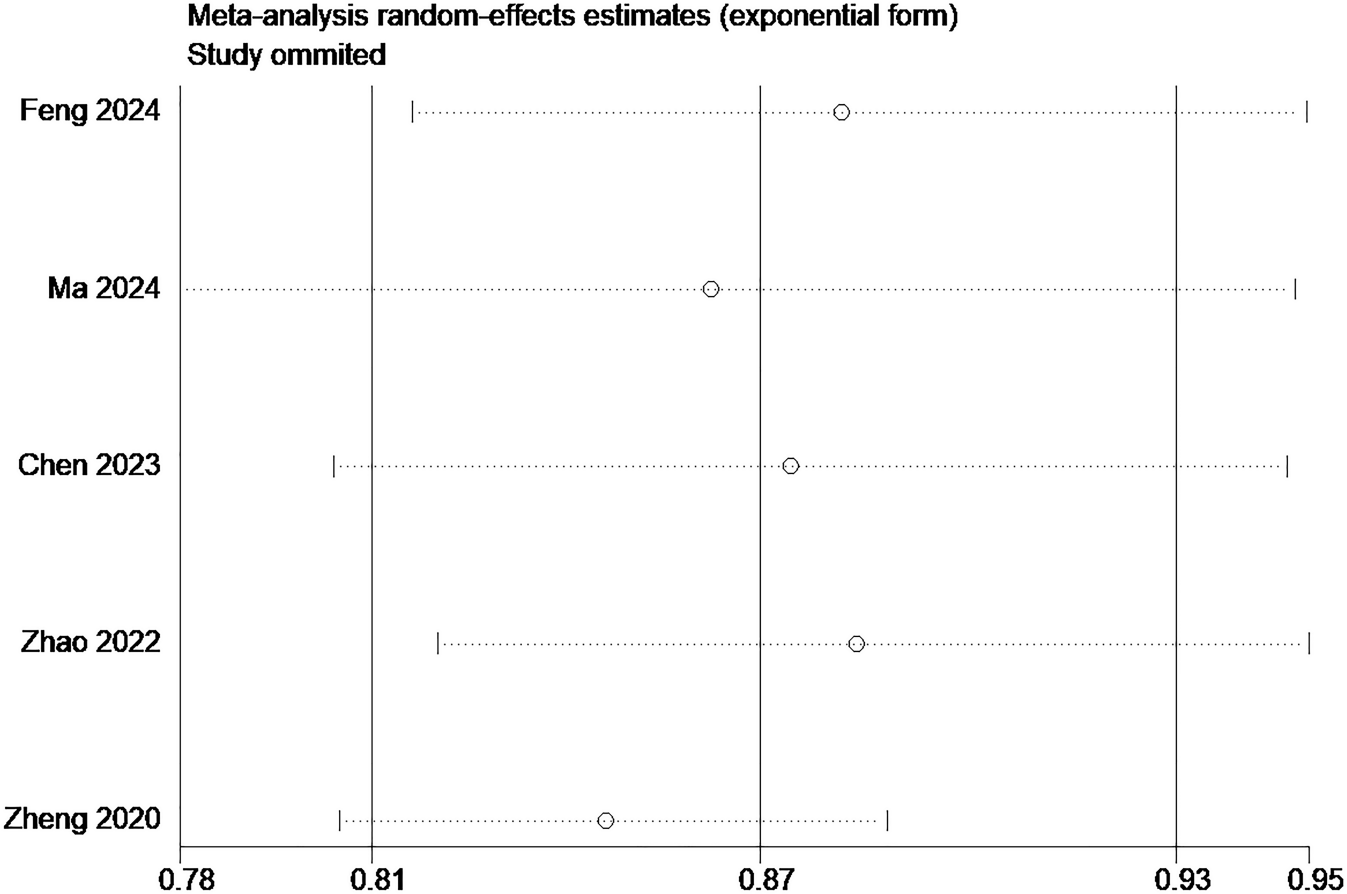
Results of the sensitivity analysis of the 5 studies.

Among the included studies, five studies (17–19, 21, 22) were selected for the meta-analysis based on the following criteria (1): detailed reporting of model development, including the use of appropriate statistical methods (2); the availability of complete AUC values and 95% confidence intervals. These criteria were used to ensure the robustness and comparability of the models included in the meta-analysis. A random effects model was used to combine the above five studies, and the combined AUC value was 0.868 (95% confidence interval: 0.810-0.929) (**Figure 3**). The calculated I² value was 86.6%(*p*<0.001), indicating high heterogeneity across studies. Further, Egger’s test results showed that the value was -2.31 (*p* =0.104), suggesting no obvious publication bias in this study. **Figure 4** displays the results of the sensitivity analysis. From the figure, it can be observed that after excluding any single study, the estimated AUC values remain around 0.87, and the error bars are relatively short, indicating that the predictive power of these models is quite stable.

## 4 Discussion

### 4.1 The quality of studies on risk prediction models for chemotherapy-related mucositis in malignant tumour patients is acceptable, but certain biases exist.

The 11 included prediction models demonstrated good overall predictive performance, with most AUCs clustering in the higher range. However, the high heterogeneity among these models may be attributable to the differences in population, predictors, and methodology across the various models. ​​Quantitative analysis based on the PROBAST tool identified significant biases in the analysis domain (1): six studies (17–22) had an events-per-variable (EPV) ratio <20, substantially increasing the risk of overfitting (2); four studies (18, 20, 21, 23) employed univariate predictor selection, which may introduce bias by ignoring multicollinearity and interactions among variables (3); only four studies (17, 20, 21, 24) reported external validation, and none assessed temporal or spatial validation, limiting the generalizability across different settings and periods. These biases, as quantified by PROBAST, primarily manifested as insufficient outcome events (EPV <20), suboptimal variable selection methods (univariate analysis), and inadequate validation protocols, which collectively contributed to the high overall risk of bias concentrated in the analysis domain.​

#### 4.1.1 In terms of data sources

The six studies (17–22) included in this analysis are retrospective case-control studies with relatively small sample sizes. It has been demonstrated that prospective studies offer better data representativeness. In contrast, retrospective studies utilising existing data may not adequately fulfil the requirements for model construction, potentially leading to recall bias and affecting their overall quality (26). In prospective studies, the measurement of predictors occurs before the outcome, effectively unifying the assessment methods of predictors and, to some extent, enhancing the reliability of the model results. The PROBAST evaluation tool (27) indicates that to reduce overfitting in model development studies, the number of outcome events should be 20 times the number of candidate predictors; that is, the number of events per variable (EPV) should exceed 20. Given the numerous candidate predictors in the prediction model for chemotherapy-related mucositis in patients with malignant tumours, meeting the EPV > 20 requirement is challenging. Furthermore, most models utilise the WHO oral mucositis assessment scale as the diagnostic basis for chemotherapy-related oral mucositis. This scale primarily relies on the subjective observations of nursing staff regarding patients’ symptoms of oral mucositis, such as erythema and ulcers, and lacks objective quantitative indicators. Consequently, this may lead to discrepancies in the assessment results of the same patient by different nursing staff, thereby affecting the reliability and consistency of the evaluation (28).

Future research should prioritise adopting prospective study designs to reduce recall bias and ensure that predictor measurements occur before outcomes, thereby enhancing data quality and model reliability. Concurrently, the sample size and number of events should be increased to meet the requirement of at least a 20:1 ratio of events to candidate predictors (EPV > 20). This can be achieved through multicentre collaboration to expand the sample size and improve the representativeness of the study findings. Additionally, assessment tools need to be optimised by incorporating more objective quantitative measures and integrating patient self-assessment tools to enhance the accuracy and comprehensiveness of evaluations. Assessment standards should be standardised throughout the research process, and data verification and cleaning should be strengthened to minimise data bias. Furthermore, advanced statistical methods, such as cross-validation and regularisation, can be employed to mitigate the risk of model overfitting. Special attention should be given to the unique needs of specific patient populations, and individualised prediction models should be developed to enhance both the specificity and effectiveness of the models.

#### 4.1.2 In terms of design and construction

The WHO-OM scale has certain limitations in assessing the dynamic changes of oral mucositis. Due to its relatively simple grading criteria, it is challenging to accurately reflect the subtle changes in patients’ conditions during the treatment process, hampers the timely adjustment of treatment and nursing plans by healthcare providers. In this study, all models were static, allowing for calculating incidence rates, but lacking a dynamic assessment of changes in patients’ health status. Most studies in this research employed logistic regression to construct models, assigning values to predictive factors based on regression coefficient weights to calculate risk. However, the correlation between variables is difficult to avoid, resulting in significant heterogeneity affecting the models’ accuracy and practicality. Machine learning has emerged as a focal point in analysing disease-influencing factors, utilising various algorithms to enhance the predictive capability of models in addressing complex issues.

Nevertheless, machine learning is hindered by challenges such as poor interpretability and cumbersome computations. On et al. (23) developed predictive models using three machine learning algorithms: logistic regression, decision trees, and artificial neural networks, to predict eight types of chemotherapy-induced adverse drug reactions (ADRs). By comparing the performance of different algorithms, the study revealed that the logistic regression model excelled in predicting six types of ADRs. At the same time, decision trees and artificial neural networks exhibited good predictive capabilities for specific ADRs.

Therefore, optimising logistic regression algorithms, developing interpretable machine learning algorithms, and enhancing model performance are important directions for future research. Additionally, integrating model presentation with artificial intelligence to simplify manual calculations and improve the accuracy and operability of models is crucial. Furthermore, the models included in this study did not refine the disease risk stratification. It is recommended that future research categorise the probability of disease occurrence into high-risk, medium-risk, low-risk, and very low-risk levels, thereby enabling clinical practices to adopt targeted measures based on these different risk levels.

#### 4.1.3 In terms of statistical analysis

Four studies (17, 20–22) incorporated statistically significant variables into their models based on univariate analysis. Although this method of screening predictors can reduce workload, it may overlook important risk factors. In variable selection, it is crucial to integrate professional knowledge and clinical experience, carefully incorporating and removing variables (29). When a large number of candidate variables are identified, researchers can utilise Lasso regression to eliminate irrelevant variables, which aids in identifying key factors within the dataset, reduces model complexity, avoids overfitting, and enhances the model’s predictive accuracy (30). None of the six studies (17–22) reported missing data, with only one study (18) opting for multiple imputation to address this issue. The advantage of this method lies in its consideration of various uncertainties arising during the imputation process of missing data, allowing for inference on the unknown characteristics of the missing data while effectively maintaining the relationships between variables (31). Additionally, two other studies employed assumed imputation and the K-nearest neighbours (KNN) method for filling in missing data, respectively. Assumed imputation, based on statistical assumptions, is suitable for large-scale datasets, as it can reduce bias and preserve the overall statistical properties of the data. In contrast, the KNN imputation method, which is based on similarity, is more appropriate for complex data structures, better retaining the local structure and similarity of the data. We also recommend that researchers meticulously document the reasons for and the extent of missing data to enhance the robustness of the models.

In future research, methods for handling missing data should be selected based on the data’s specific characteristics and the research needs. Three studies (21–23) did not report the calibration of their models, while four studies (18, 19, 22, 23) failed to conduct internal or external validation. It is recommended that future research on predictive models refer to PROBAST and the Transparent Reporting of a Multivariable Prediction Model for Individual Prognosis or Diagnosis (TRIPOD) (32) to guide model design and reporting, thereby controlling for model bias risks and standardising reporting practices. ​​Given that only four studies (17, 20, 21, 24) reported external validation with relatively small sample sizes, this raises concerns about the generalizability of the models to real-world clinical settings. Future research should prioritise prospective, multicentre external validation studies and explore the development of dynamic prediction tools—for instance, by incorporating longitudinal data (e.g., repeated assessments of oral mucositis status during chemotherapy cycles) to capture time-dependent disease progression and improve clinical applicability.​​ Given the differences in research locations and subjects, prediction models must undergo internal and external validation before being applied in clinical settings to mitigate overfitting and ensure their applicability and effectiveness. However, in this study, only 38% (3/8) of the models employed a combined internal and external validation approach, and over 50% did not undergo external validation, which limits their extrapolability. External validation is essential for assessing the stability and effectiveness of models and is more time- and cost-efficient than rebuilding models. Therefore, future research could build on this study by selecting high-quality models for optimisation and calibration, utilising spatial and temporal validation methods to enhance model performance.

### 4.2 Selection commonality of predictors

The six models in this study contained potential predictors ranging from 12 to 28, with the final models comprising 4 and 7 predictors. Due to variations in study types and included variables, the top five consistently reported predictors were age, oral hygiene, smoking history, chemotherapy cycles, and chemotherapy regimen. CaKmak et al. (33) reported that elderly patients were 2.03 times more likely to develop oral mucositis than their younger counterparts. Elderly patients experience reduced metabolism and decreased resistance, rendering their mucosa more susceptible to infection. The oral mucosa functions as a barrier and defence against external bacterial invasion. Poor oral hygiene, characterised by food debris lodged in the mouth’s crevices, can lead to the proliferation of oral parasitic bacteria, thereby damaging the oral mucosa.

Additionally, reduced water and food intake by patients weakens the self-cleaning function of the mouth, resulting in oral mucositis (34). Therefore, it is recommended that clinical nursing staff pay close attention to the impact of oral cleanliness on the oral mucosa of patients with malignant tumours, particularly focusing on the oral hygiene of elderly and uncooperative patients. Prolonged smoking exposes the oral mucosa to toxic substances in tobacco, such as phenols, which irritate the mucosa. The high temperature of the mouth during smoking can cause burns to the contact areas of the oral mucosa. Smoking can also affect local blood circulation, impair humoral immunity, interfere with tissues at the cellular level, and impact tissue metabolism (35). Studies have shown that smoking is a risk factor for chemotherapy-induced oral mucositis, with the incidence of oral mucositis in smoking patients being significantly higher than in non-smoking patients after chemotherapy (36). While our analysis identified smoking as a risk factor, the included studies did not stratify patients by smoking status (current, former, or never smokers). This omission limits insights into whether active smoking during chemotherapy, cumulative tobacco exposure, or smoking cessation impacts CIOM severity. For instance, chronic smoking may damage oral mucosal nociceptive fibres, paradoxically reducing opioid requirements despite severe mucositis. A study by Murphy et al. (37) demonstrated that HPV-positive oropharyngeal cancer patients with a history of heavy smoking reported lower opioid consumption during chemoradiation-induced mucositis compared to HPV-negative patients, suggesting neuropathic changes from long-term tobacco use may attenuate pain perception. This phenomenon highlights the need to distinguish between acute and chronic smoking effects in CIOM risk models. Future studies should incorporate granular smoking data (e.g., pack-years, cessation duration) and assess nociceptive fibre integrity to clarify these relationships. The occurrence of oral mucositis has a cumulative effect. Combination therapy (e.g., paclitaxel and platinum) enhances the cytotoxicity against gynaecological tumour cells; however, the associated side effects can also produce a synergistic effect. There is a risk of oral mucositis with each chemotherapy cycle. Generally, if a patient develops mucositis during the first treatment cycle, the likelihood of recurrence in subsequent cycles is high without a dose reduction (38). Currently, multiple chemotherapy drugs are frequently combined in cancer treatment, complicating the identification of the specific chemotherapy agent most relevant to the occurrence of oral mucositis. The findings from Curra et al. (39) indicate that patients treated with a combination of methotrexate, cyclophosphamide, and/or doxorubicin have an increased risk of developing oral mucositis, with the risk escalating alongside higher drug doses and longer treatment cycles.

Therefore, when the chemotherapy regimen includes methotrexate or a combination of multiple chemotherapeutic agents, it is essential to enhance oral hygiene education for patients undergoing such treatment. This education should explain the potential damage these drugs can cause to the oral mucosa, raising awareness among patients and their families. Patients should be advised to increase their water intake during chemotherapy and consume foods rich in vitamins and proteins. Children should be encouraged to perform daily oral care to maintain oral cleanliness, thereby preventing and reducing the occurrence of CIOM. In addition to the repeatedly reported predictors, the other predictors identified by each model vary significantly. The analysis suggests that these differences are primarily related to the varying disease distributions among the study populations included in each model. It is recommended that independent predictive models for different tumour types be developed and appropriate and widely-used assessment scales be selected to evaluate outcome events based on disease types, enhance model stability, and promote the clinical translation of these models.

### 4.3 Potential influencing factors

While age, oral hygiene, smoking history, chemotherapy cycles, and regimens emerged as consistent predictors of CIOM, the potential role of viral co-infections in virus-related malignancies warrants further exploration. Viruses such as Epstein-Barr virus (EBV), human papillomavirus (HPV), and herpes simplex virus (HSV) are implicated in specific cancers (e.g., nasopharyngeal carcinoma, HPV-associated oropharyngeal tumours). They may exacerbate mucosal damage through immune modulation or direct cytopathic effects (40, 41). However, none of the included studies explicitly incorporated viral status as a predictor, likely due to heterogeneous study populations (lacking stratification by viral-associated malignancies), limited routine collection of virological data in retrospective cohorts, or statistical insignificance in small samples. For instance, Curra et al. (39) observed higher CIOM incidence in paediatric haematological malignancies—a population prone to viral reactivation—though viral co-infections were not systematically analysed. Similarly, immunosuppression, identified as a risk factor by Çakmak and Nural (33), may indirectly reflect viral contributions, as viral reactivation in immunocompromised patients can amplify mucosal inflammation.

### 4.4 Development trends and challenges of risk prediction models for chemotherapy-induced oral mucositis in malignant tumour patients

Among the included studies, only two were conducted in multiple centres (20, 24). Future research could be expanded to include multicentre, large-sample application studies to enhance the replicability of the models and promote their implementation. The internal validation of the risk prediction models for chemotherapy-induced oral mucositis (CIOM) in malignant tumour patients demonstrated good predictive capability; however, external validation is lacking. Only three articles (17, 20, 24) conducted internal and external validations, with a small external validation sample size. Future research should prioritise large-sample external validation to assess the transferability and extrapolation of the models. Healthcare professionals could also leverage data mining techniques to thoroughly explore factors associated with the occurrence of CIOM, thereby improving the accuracy of the prediction models (42). In the future, when healthcare professionals apply predictive models in clinical practice, they should focus on timely optimisation and continuous calibration of these models by integrating the individual characteristics of high-risk populations. By conducting early screening and identifying high-risk populations and risk factors, the occurrence of CIOM can be predicted, which assists nursing staff in providing appropriate nursing interventions for high-risk populations. This approach ensures positive patient outcomes, alleviates the economic burden on patients, and reduces medical costs.

## 5 Limitations

This systematic review has several limitations:①Only studies published in Chinese and English were included, which may introduce publication bias; ②All the included studies were found to be at high risk of bias after quality assessment. This high risk of bias may be attributed to inadequate sample size, lack of randomisation, and incomplete outcome data. These biases may have affected the validity and reliability of the results, and thus, the conclusions drawn from this systematic review should be interpreted with caution.

## 6 Conclusion

This systematic review confirms that the included prediction models achieve robust discriminative performance. However, the current prediction models for chemotherapy-induced mucositis in cancer patients do not meet the PROBAST criteria. Nevertheless, the predictors consistently reported by these models can provide valuable references for preventing and managing chemotherapy-induced oral mucositis (CIOM). In the future, researchers should focus on screening candidate variables that are easily accessible, accurately measurable, and low in measurement cost, while prioritising the development of new models with larger sample sizes, rigorous study designs, and multicentre external validation for various disease types.

## Data Availability

The original contributions presented in the study are included in the article/**Supplementary Material**. Further inquiries can be directed to the corresponding author.
