# Supplementary material for: Predictive models for chemotherapy-induced oral mucositis: a systematic review
Source: Front Oncol. 2025 Aug 20;15:1608505. doi: 10.3389/fonc.2025.1608505 (PMC12404939; doi:10.3389/fonc.2025.1608505)
Supplement: Supplementary file 1 [file DataSheet1.docx]

**Search ran: 2025.5.9**

**Table S1. Search terms in databases**

| **Databases** | **Step** | **Searches** | **Results** |
| --- | --- | --- | --- |
| PubMed | #1 | "Stomatitis"[MeSH Terms] OR "Stomatitis"[MeSH Terms] OR "Stomatitis"[All Fields] OR "stomatitides"[All Fields] OR "oral mucositis"[All Fields] OR "oral cavity"[All Fields] OR "oral mucosa"[All Fields] OR "oral muco*"[All Fields] OR "oral complications"[All Fields] OR "oral mucosa injuries"[All Fields] OR "Stomatitis"[MeSH Terms] OR "Stomatitis"[All Fields] OR "oromucositis"[All Fields] OR "OM"[All Fields] | 129,573 |
|  | #2 | "Neoplasms"[MeSH Terms] OR ("neoplas*"[All Fields] OR ("cancer s"[All Fields] OR "cancerated"[All Fields] OR "canceration"[All Fields] OR "cancerization"[All Fields] OR "cancerized"[All Fields] OR "cancerous"[All Fields] OR "Neoplasms"[MeSH Terms] OR "Neoplasms"[All Fields] OR "cancer"[All Fields] OR "cancers"[All Fields]) OR ("cysts"[MeSH Terms] OR "cysts"[All Fields] OR "cyst"[All Fields] OR "neurofibroma"[MeSH Terms] OR "neurofibroma"[All Fields] OR "neurofibromas"[All Fields] OR "tumor s"[All Fields] OR "tumoral"[All Fields] OR "tumorous"[All Fields] OR "tumour"[All Fields] OR "Neoplasms"[MeSH Terms] OR "Neoplasms"[All Fields] OR "tumor"[All Fields] OR "tumour s"[All Fields] OR "tumoural"[All Fields] OR "tumourous"[All Fields] OR "tumours"[All Fields] OR "tumors"[All Fields]) OR ("carcinoma"[MeSH Terms] OR "carcinoma"[All Fields] OR "carcinomas"[All Fields] OR "carcinoma s"[All Fields]) OR ("malign"[All Fields] OR "malignance"[All Fields] OR "malignances"[All Fields] OR "malignant"[All Fields] OR "malignants"[All Fields] OR "malignities"[All Fields] OR "malignity"[All Fields] OR "malignization"[All Fields] OR "malignized"[All Fields] OR "maligns"[All Fields] OR "Neoplasms"[MeSH Terms] OR "Neoplasms"[All Fields] OR "malignancies"[All Fields] OR "malignancy"[All Fields]) OR ("malign"[All Fields] OR "malignance"[All Fields] OR "malignances"[All Fields] OR "malignant"[All Fields] OR "malignants"[All Fields] OR "malignities"[All Fields] OR "malignity"[All Fields] OR "malignization"[All Fields] OR "malignized"[All Fields] OR "maligns"[All Fields] OR "Neoplasms"[MeSH Terms] OR "Neoplasms"[All Fields] OR "malignancies"[All Fields] OR "malignancy"[All Fields]) OR ("metastasi"[All Fields] OR "neoplasm metastasis"[MeSH Terms] OR ("neoplasm"[All Fields] AND "metastasis"[All Fields]) OR "neoplasm metastasis"[All Fields] OR "metastasis"[All Fields]) OR ("metastasation"[All Fields] OR "metastasic"[All Fields] OR "metastasing"[All Fields] OR "metastasise"[All Fields] OR "metastasised"[All Fields] OR "metastasises"[All Fields] OR "metastasising"[All Fields] OR "metastasization"[All Fields] OR "metastasizes"[All Fields] OR "metastasizing"[All Fields] OR "neoplasm metastasis"[MeSH Terms] OR ("neoplasm"[All Fields] AND "metastasis"[All Fields]) OR "neoplasm metastasis"[All Fields] OR "metastase"[All Fields] OR "metastases"[All Fields] OR "metastasize"[All Fields] OR "metastasized"[All Fields])) | 6,164,075 |
|  | #3 | "Drug Therapy"[MeSH Terms] OR ("chemotherapy s"[All Fields] OR "Drug Therapy"[MeSH Terms] OR ("drug"[All Fields] AND "therapy"[All Fields]) OR "Drug Therapy"[All Fields] OR "chemotherapies"[All Fields] OR "Drug Therapy"[MeSH Subheading] OR "chemotherapy"[All Fields] OR "chemotherap*"[All Fields] OR "chemical therapy"[All Fields] OR ("chemotherapeutic"[All Fields] OR "chemotherapeutical"[All Fields] OR "chemotherapeutically"[All Fields] OR "chemotherapeutics"[All Fields]) OR "antineoplastic agent"[All Fields] OR "antineoplastic drug"[All Fields]) | 4,236,171 |
|  | #4 | "Risk Assessment"[MeSH Terms] OR "predict* model"[All Fields] OR "prognos* model"[All Fields] OR "risk predict*"[All Fields] OR "risk score"[All Fields] OR "predictive factor*"[All Fields] OR "risk factor*"[All Fields] | 1,835,290 |
|  | #5 | #1 AND #2 AND #3 AND #4 | 855 |

| **Databases** | **Step** | **Searches** | **Results** |
| --- | --- | --- | --- |
| Web of science | #1 | ["Stomatitis" (Topic) or stomatitiden (Topic) or "oral mucositis" (Topic) or "oral cavity" (Topic) or "oral mucosa" (Topic) or “oral muco*” (Topic) or "oral complications" (Topic) or "oral mucosa injuries" (Topic) or oralmucositis (Topic) or OM (Topic)](https://webofscience.clarivate.cn/wos/alldb/summary/cc6a27c0-27fe-4e70-b8fb-c6779719acaf-0161b685a5/relevance/1) | 192,291 |
|  | #2 | Neoplasms (Topic) OR “neoplas*” (Topic) OR cancer (Topic) OR tumor (Topic) OR carcinoma (Topic) OR malignancy (Topic) OR malignancies (Topic) OR metastasis (Topic) OR metastases (Topic) | 8,310,212 |
|  | #3 | "Drug Therapy" (Topic) OR chemotherapy (Topic) OR “chemotherap*” (Topic) OR “chemical therapy” (Topic) OR chemotherapeutic (Topic) OR “antineoplastic agent” (Topic) OR “antineoplastic drug” (Topic) | 5,509,418 |
|  | #4 | "Risk Assessment" (Topic) or “predict* model” (Topic) or “prognos* model” (Topic) or “risk predict*” (Topic) or “risk score” (Topic) or “predictive factor*” (Topic) or “risk factor*” (Topic) | 2,730,494 |
|  | #5 | #1 AND #2 AND #3 AND #4 | 1527 |

| **Databases** | **Step** | **Searches** | **Results** |
| --- | --- | --- | --- |
| Cochrone | #1 | [Stomatitis] OR (stomatitides):ti,ab,kw OR (oral mucositis):ti,ab,kw OR (oral cavity):ti,ab,kw OR (oral mucosa):ti,ab,kw OR (oral muco*):ti,ab,kw OR (oral complications):ti,ab,kw OR (oral mucosa injuries):ti,ab,kw OR (oromucositis):ti,ab,kw OR (OM):ti,ab,kw | 34812 |
|  | #2 | [Neoplasms] OR (neoplas*):ti,ab,kw OR (cancer):ti,ab,kw OR (tumor):ti,ab,kw OR (carcinoma):ti,ab,kw OR (malignancy):ti,ab,kw OR (malignancies):ti,ab,kw OR (metastasis):ti,ab,kw OR (metastases):ti,ab,kw | 296610 |
|  | #3 | [Drug Therapy] OR (chemotherapy):ti,ab,kw OR (chemotherap*):ti,ab,kw OR (chemical therapy):ti,ab,kw OR (chemotherapeutic):ti,ab,kw OR (antineoplastic agent):ti,ab,kw OR (antineoplastic drug):ti,ab,kw | 262582 |
|  | #4 | [Risk Assessment] OR (predict* model):ti,ab,kw OR (prognos* model):ti,ab,kw OR (risk predict*):ti,ab,kw OR (risk score):ti,ab,kw OR (predictive factor*):ti,ab,kw OR (risk factor*):ti,ab,kw | 112565 |
|  | #5 | #1 AND #2 AND #3 AND #4 | 287 |

| **Databases** | **Step** | **Searches** | **Results** |
| --- | --- | --- | --- |
| Embase | #1 | 'stomatitis'/exp OR stomatitides OR 'oral mucositis' OR 'oral cavity' OR 'oral mucosa' OR 'oral muco*' OR 'oral complications' OR 'oral mucosa injuries' OR oromucositis OR om | 188,026 |
|  | #2 | 'neoplasm'/exp OR 'neoplas*' OR cancer OR tumor OR carcinoma OR malignancy OR malignancies OR metastasis OR metastases | 8.707.281 |
|  | #3 | 'drug therapy'/exp OR chemotherapy OR 'chemotherap*' OR 'chemical therapy' OR chemotherapeutic OR 'antineoplastic agent' OR 'antineoplastic drug' | 4.689,192 |
|  | #4 | 'risk assessment'/exp OR 'predict* model' OR 'prognos* model' OR 'risk predict*' OR 'risk score' OR 'predictive factor*' OR 'risk factor*' | 2,721,105 |
|  | #5 | #1 AND #2 AND #3 AND #4 | 3.041 |

| **Databases** | **Searches** | **Results** |
| --- | --- | --- |
| 知网 | （主题：口腔黏膜炎 + 口腔粘膜炎 + 口炎 + 口腔炎+口腔溃疡 + 口腔感染 + 口疮 + 口腔黏膜反应）AND（主题：癌症 + 肿瘤 + 恶性肿瘤）AND（主题：化疗 + 化学疗法 + 化学治疗 + 化学药物治疗）AND（主题：风险预测 + 风险预测模型 + 预测模型 + 诊断模型 + 风险评分 + 风险评估 + 临床预测模型 + 风险因素 + 列线图） | 20 |
| 万方 | 题名或关键词:(口腔黏膜炎 OR 口腔粘膜炎 OR 口炎 OR 口腔炎 OR 口腔溃疡 OR 口腔感染 OR 口疮 OR 口腔黏膜反应) and 题名或关键词:(化疗 OR 化学疗法 OR 化学治疗 OR 化学药物治疗) and 题名或关键词:(癌症 OR 肿瘤 OR 恶性肿瘤) and 题名或关键词:(风险预测 OR 风险预测模型 OR 预测模型 OR 诊断模型 OR 风险评分 OR 风险评估 OR 临床预测模型 OR 风险因素 OR 列线图) | 7 |
| 维普 | ((((((((((题名或关键词=口腔黏膜炎 OR 题名或关键词=口腔粘膜炎) OR 题名或关键词=口炎) OR 题名或关键词=口腔炎) OR 题名或关键词=口腔溃疡) OR 题名或关键词=口腔感染) OR 题名或关键词=口疮) OR 题名或关键词=口腔黏膜反应) AND (((题名或关键词=化疗 OR 题名或关键词=化学疗法) OR 题名或关键词=化学治疗) OR 题名或关键词=化学药物治疗)) AND ((题名或关键词=癌症 OR 题名或关键词=肿瘤) OR 题名或关键词=恶性肿瘤)) AND ((((((((题名或关键词=风险预测 OR 题名或关键词=风险预测模型) OR 题名或关键词=预测模型) OR 题名或关键词=诊断模型) OR 题名或关键词=风险评分) OR 题名或关键词=风险评估) OR 题名或关键词=临床预测模型) OR 题名或关键词=风险因素) OR 题名或关键词=列线图)) | 2 |
| 中国生物医学数据库 | (("化疗"[常用字段:智能] OR "化学疗法"[常用字段:智能] OR "化学治疗"[常用字段:智能] OR "化学药物治疗"[常用字段:智能]) OR ("药物疗法"[不加权:扩展])) AND (("癌症"[常用字段:智能] OR "恶性肿瘤"[常用字段:智能]) OR ("肿瘤"[不加权:扩展])) AND ((("口腔黏膜炎"[常用字段:智能] OR "口腔粘膜炎"[常用字段:智能] OR "口腔炎"[常用字段:智能] OR "口腔溃疡"[常用字段:智能] OR "口腔感染"[常用字段:智能] OR "口疮"[常用字段:智能] OR "口腔黏膜反应"[常用字段:智能]) AND -2025[日期]) OR ("口炎"[不加权:扩展])) AND ("风险预测"[常用字段:智能] OR "风险预测模型"[常用字段:智能] OR "预测模型"[常用字段:智能] OR "诊断模型"[常用字段:智能] OR "风险评分"[常用字段:智能] OR "风险评估"[常用字段:智能] OR "临床预测模型"[常用字段:智能] OR "风险因素"[常用字段:智能] OR "列线图"[常用字段:智能]) | 23 |
